# Prognostic significance of tumor regression grade in esophageal squamous cell carcinoma after neoadjuvant chemoradiation

**DOI:** 10.3389/fsurg.2022.1029575

**Published:** 2023-01-06

**Authors:** Chi Zhang, Fei Xu, Yong Qiang, Zhuang-Zhuang Cong, Qin Wang, Zheng Zhang, Chao Luo, Bing-Mei Qiu, Li-Wen Hu, Yi Shen

**Affiliations:** ^1^ Department of Cardiothoracic Surgery, Jinling Hospital, Medical School of Nanjing University, Nanjing, China; ^2^ Department of Cardiothoracic Surgery, Jinling Hospital, School of Clinical Medicine, Nanjing Medical University, Nanjing, China; ^3^ Department of Cardiothoracic Surgery, Jinling Hospital, School of Medicine, Southeast University, Nanjing, China; ^4^ Department of Cardiothoracic Surgery, Jinling Hospital, School of Clinical Medicine, Southern Medical University, Guangzhou, China; ^5^ Department of Cardiothoracic Surgery, Jinling Hospital, Nanjing, China

**Keywords:** esophageal squamous cell carcinoma, neoadjuvant chemoradiotherapy, esophagectomy, tumor regression grade, modified ryan scoring system

## Abstract

**Backgrounds:**

Trimodal therapy (neoadjuvant chemoradiotherapy followed by esophagectomy) for locally advanced esophageal squamous cell carcinoma (ESCC) is associated with a significant survival benefit. Modified Ryan score is an effective tool to evaluated the tumor regression grade (TRG) after neoadjuvant therapy. The aim of this study was to evaluate the prognostic value of TRG for overall survival (OS) and disease-free survival (DFS) in ESCC patients undergoing neoadjuvant chemoradiation.

**Methods:**

The study retrospectively reviewed 523 ESCC patients who underwent neoadjuvant chemoradiotherapy and radical esophagectomy at Jinling Hospital from January 2014 to July 2020. Kaplan–Meier curves with log-rank test and Cox regression model were used to evaluate the prognostic factor of TRG based on modified Ryan scoring system on OS and DFS.

**Results:**

After application of inclusion and exclusion criteria, 494 patients with ESCC following neoadjuvant chemoradiotherapy and radical esophagectomy were available for analysis. The TRG scores are significantly associated with smoke history (*p* = 0.02), lymphovascular invasion (LVI) and/or peripheral nerve invasion (PNI) (*p* < 0.01), and postoperative adjuvant therapy (*p* < 0.01). Meanwhile, tumor characteristics including tumor length (*p* < 0.01) and tumor differentiation grade (*p* < 0.01) are also significantly associated with TRG score. The results of multivariable Cox regression modal showed that TRG is not an independently prognostic factor for OS (*p* = 0.922) or DFS (*p* = 0.526) but tumor length is an independently prognostic factor for DFS (*p* = 0.046).

**Conclusions:**

This study evaluated the prognostic value of modified Ryan scoring system for ESCC after trimodal therapy and concluded that modified Ryan scoring system can predict survival and recurrence rates but is not an independently prognostic factor for OS and DFS.

## Introduction

Esophageal cancer (EC) is now the sixth leading cause of cancer deaths worldwide and the second deadliest gastrointestinal cancer after gastric carcinoma (1). The morbidity of EC varies extremely from areas and countries. Literatures reported that about 200,000 people die of EC annually worldwide and most cases of EC are diagnosed at an advanced stage (2). Esophageal squamous cell carcinoma (ESCC) is the most common EC in China. Although tremendous improvement of therapeutic modalities has been seen recently, the ESCC patient's quality of life remains poor and the 5-year survival rate rarely exceeds 40% (1). Currently, the standard treatment for clinical stages I/II/III (except for T4) ESCC is based on a combination of esophagectomy with/without adjuvant with/without neoadjuvant chemotherapy or chemoradiotherapy (3). Relative to surgery alone, multimodality therapy for locally advanced disease is associated with a significant survival benefit. It has been reported that EC patients could benefit from neoadjuvant therapy, and thus the standard treatment for these patients is neoadjuvant therapy followed by surgery (4).

The long-term survival after esophagectomy with neoadjuvant chemoradiotherapy is primarily based on the neoadjuvant treated TNM (ypTNM) staging according to the eighth American Joint Committee on Cancer (AJCC) staging system for esophageal cancer (5). However, the tumor characteristics generally are not used for prognosis. Neither tumor characteristics, such as tumor length, tumor histology, or tumor differentiation grade, nor tumor regression grade (TRG) are incorporated in the 8^th^ AJCC ypTNM staging (6). The number of ESCC patients undergoing neoadjuvant chemoradiotherapy followed by surgery has been increasing, and it is necessary to explore which pathological factors in addition to ypTNM might be associated with an overall survival (OS) and disease-free survival (DFS).

The influence of the tumor length and tumor differentiation of EC on survival has been assessed in ESCC or mixed cohorts with ESCC and esophageal adenocarcinoma (EAC) (7, 8). Generally, patients with a shorter tumor length and a favorable tumor differentiation grade have a better long-term survival than patients with adverse tumor characteristics. A number of TRG scoring systems are used to assess the effectiveness of neoadjuvant therapy (9). One of these is the Ryan scoring system, based on the ratio of residual cancer cells to the amount of fibrosis (10). The Ryan scoring system ranges from 1 (complete or near-complete response) to 3 (poor or not response to neoadjuvant therapy). The reproducibility and prognostic value of Ryan scoring system were extensively studied in a variety of cancers, in which Ryan scoring system has been proved to be a reliable instrument to classify the tumor regression (9, 11). Modified Ryan scoring system was subsequently introduced to divide score 1 into two group: score 0 (complete response) and score 1 (near-complete response), which was more precise to stratify the patients undergoing neoadjuvant therapy compared with Ryan scoring system (11).

Accordingly, the 8^th^ AJCC considers TRG an additional prognostic factor for rectal cancers after neoadjuvant therapy but failed to add this into the staging system (12, 13). However, whether TRG graded based on modified Ryan scoring system could be considered as a prognostic factor in addition to ypTNM in patients undergoing neoadjuvant chemoradiotherapy and esophagectomy remains controversial. Therefore, we performed this large-scale retrospective study to evaluate the independent relationship of post-treatment pathologic regression with OS and DFS in ESCC.

## Methods

### Patients

The study retrospectively reviewed 523 ESCC patients who underwent neoadjuvant chemoradiotherapy and radical esophagectomy at Jinling Hospital from January 2014 to July 2020. This study was approved by Jinling Hospital institutional review board. All the patients were informed concerning the risks of the neoadjuvant/adjuvant therapy and esophagectomy.

**Figure 1:**
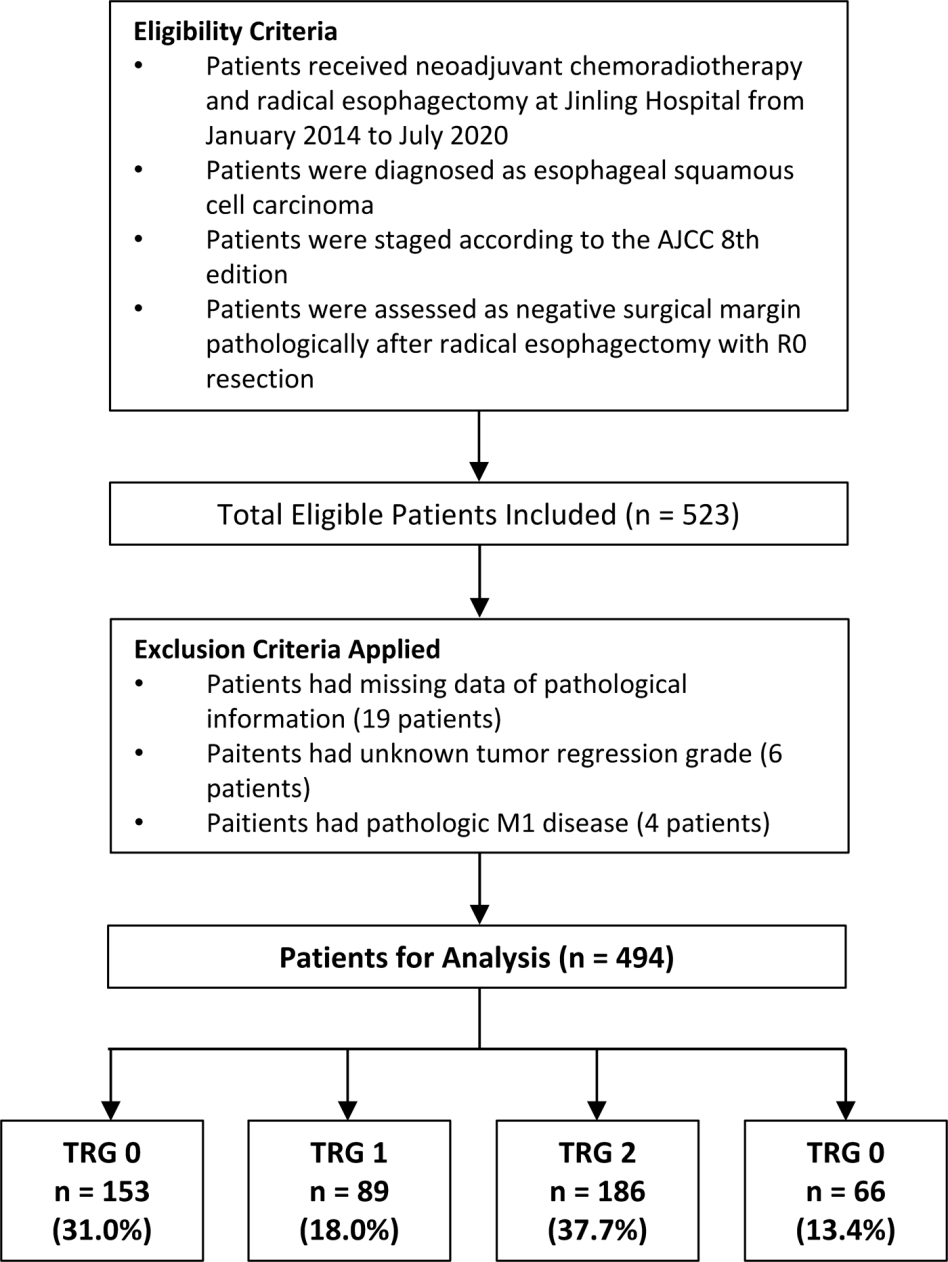
CONSORT diagram.

The inclusion criteria are listed as follows: (1) patients pathologically were diagnosed as ESCC before treatment; (2) patients received neoadjuvant chemoradiotherapy and esophagectomy; (3) patients were staged according to the American Joint Committee on Cancer (AJCC) 8^th^ edition (5) (5); detailed data on the pathological information and tumor regression grade were collected (6); patients were assessed as negative surgical margin pathologically after radical esophagectomy with R0 resection. Patients were excluded if they: had missing data of pathological information, had unknown tumor regression grade, or had pathologic M1 disease. The CONSORT diagram (Figure 1) shows the inclusion and exclusion criteria of our study.

### Tumor regression grade

We referred to the modified Ryan scoring system to score tumor regression grades (TRGs) (10). The TRG 0–3 are defined as follows: TRG 0: no viable cancer cells (complete response); TRG 1: single cell or rare small groups of cancer cells (near complete response); TRG 2: residual cancer with evident tumor regression but more than single cell or rare small groups of cancer cells (partial response); TRG 3: extensive residual cancer with no evident tumor regression (poor or not response). Three pathologists reexamined the results of the pathological sections, and the final TRG had to be agreed upon by two or more pathologists.

Patients were divided into “TRG 0’, “TRG 1”, “TRG 2” or “TRG 3” groups for log-rank test, Kaplan–Meier analysis, and Cox regression analysis. Meanwhile, patients were further divided in to two groups (TRG 0–1 and TRG 2–3) for subgroup analysis stratified by patients' characteristics. Demographic characteristics, operative data, postoperative complications, and pathological information were collected on all patients.

### Follow-up

Patients were followed up every 3 months for the first 2 years, and then every 6 months thereafter. Neck and abdominal ultrasound, chest CT, gastroscopy, and blood test were performed on the basis of patient's symptoms during follow-up. The patient status (including death and survival), and the tumor status (including tumor recurrence and metastasis), and the patient loss of follow-up were all documented. Our follow-ups were implemented *via* telephone or outpatient department visit. The last follow-up was conducted in April 1, 2022.

### Neoadjuvant therapy

The selection of neoadjuvant therapy depended on preoperative clinical stage of EC patients. Neoadjuvant chemoradiotherapy was routinely administered for patients with cN1–3 and/or cT4a-b. Neoadjuvant chemoradiotherapy included 2 cycles of chemotherapy with sequential or concurrent radiotherapy. The neoadjuvant chemoradiotherapy treatment cycle was 3 weeks (treatment during weeks 1 and 4). Pacilitaxel in a dose of 175 mg/m^2^ (day 1) or carboplatin in a dose of AUC 5 (day 1), with a combination of cisplatin in the amount of 75 mg/m^2^/24 h (days 1–2 or days 1–3), was given intravenously. Patients received concurrent radiation to a total dose of 50 gray (Gy), delivered in 2.0 Gy per fractions, starting at day 1 of the first chemotherapy cycle (week 1) and ending at the completion of the second chemotherapy cycle (week 4). Sequential radiation to the same doses was arranged after end of the second chemotherapy cycle. Intensity-modulated radiotherapy technique was used to perform radiotherapy in all patients.

### Surgical procedure and pathology

The surgical options depended on preoperative examinations of the patients and their general condition. McKeown esophagectomy with cervical anastomoses or Ivor-Lewis esophagectomy with thoracic anastomoses combining with radical lymph node dissection were performed in a standardized manner. Meanwhile, the gastric conduit was the means of reconstruction during esophagectomy. Surgeons then separated the dissected lymph nodes from the resected esophagus and peri-esophagus tissues. Two experienced pathologists fixed the dissected specimens, then embedded and stained them with diaminobenzidine chromogen counterstain solution and hematoxylin to routinely assess resected specimens histologically and pathologically. The status of lymphovascular invasion (LVI) and peripheral nerve invasion (PNI) were also evaluated.

### Adjuvant therapy

In our institution, adjuvant therapy selection was determined by a multidisciplinary team or by patients' preference. Generally, cisplatin, taxane and/or 5-fluorouracil were included in the chemotherapy regimen. External beam radiation with a total dose of 45 to 50.4 Gy (1.8–2.0 Gy/d) was utilized to administer radiotherapy by using three-dimensional conformal radiation. Chemoradiotherapy was the radiotherapy conducted from the first day of the first chemotherapy cycle. Keytruda or Opdivo combined with radiotherapy was administrated for patients undergoing adjuvant immnoradiotherapy. Usually, adjuvant therapy started 4 to 6 weeks after surgery.

### Statistical analysis

Pearson's Chi-square tests or Fisher exact test was used to compare categorical variables expressing as frequencies. The independent-sample Student's *t*-test or the Mann-Whitney non-parametric U-test was used to compare continuous variables expressed as mean±standard deviation. Kaplan-Meier curves were used to analyze overall survival (OS) and disease-free survival (DFS), and the log-rank test was employed to determine statistical significance between groups. Cox regression model was used to determine pathologic variables independently associated with OS and DFS. Variables were selected for multivariate Cox-regression model entry if *p* < 0.05 on univariate analysis. In addition, factors with a *p*-Value < 0.05 in univariate analysis were further analyzed in a multivariate Cox proportional hazards model using a backwards model selection procedure (elimination criterion: *p* < 0.10). Finally, factors that were included in the final model were used to build the nomogram and risk classification system. All tests were two-sided, and *p* < 0.05 was considered as statistical significance. All statistical analysis was implemented with R (version 3.5.3).

## Results

### Patient characteristics

After application of inclusion and exclusion criteria, 494 patients with ESCC following neoadjuvant chemoradiotherapy and radical esophagectomy were available for analysis. Demographic characteristics, comorbidities, operative data, postoperative complications, and pathological information of included patients are displayed in Table 1. Complete response (TRG 0) was reported in 153 (31.0%) patients, near complete response (TRG 1) in 89 (18.0%) patients, partial response (TRG 2) in 186 (37.7%) patients, and poor or not response (TRG 3) in 66 (13.4%) patients. Adjuvant therapy was documented for in 159 (32.2%) patients. The tumors were graded as well and moderately differentiated (*n* = 133, 24.7%), or poorly differentiated (*n* = 161, 32.6%). For 200 patients (40.5%), the grade could not be determined (Gx). The median of tumor length was 3 cm, which was used as the cut-off value of tumor length. There were 186 (37.7%) patients having a tumor length more than 3 cm and the remaining 308 (62.3%) patients had a tumor length less than or equal to 3 cm.

**Table 1:** Patient characteristics.

Variable	All cohort No. (%) (*n* = 494)
Gender	
Male	404 (81.8%)
Female	90 (19.2%)
Missing	0 (0.0%)
Age (year)	
≤ 70	425 (86.0%)
> 70	69 (14.0%)
Missing	0 (0.0%)
Smoke	
Yes	252 (51.0%)
No	240 (48.6%)
Missing	2 (0.4%)
Tumor site	
Upper	61 (12.3%)
Middle	228 (46.2%)
Lower	205 (41.5%)
Missing	0 (0.0%)
Tumor length (cm)	
≤ 3 cm	308 (62.3%)
> 3 cm	186 (37.7%)
Missing	0 (0.0%)
ypTNM	
I	233 (47.2%)
II	75 (15.2%)
IIIA	55 (11.1%)
IIIB	116 (23.5%)
IVA	15 (3.0%)
Missing	0 (0.0%)
ypT	
T0	167 (33.8%)
T1	70 (14.2%)
T2	72 (14.6%)
T3	185 (37.4%)
Missing	0 (0.0%)
ypN	
N0	308 (62.3%)
N1	122 (24.7%)
N2	49 (9.9%)
N3	15 (3.0%)
Missing	0 (0.0%)
ypM	
M0	494 (100%)
M1	0 (0.0%)
Missing	0 (0.0%)
Tumor differentiation	
G1-2	133 (24.7%)
G3	161 (32.6%)
Gx	200 (40.5%)
Missing	0 (0.0%)
LVI and/or PNI	
Yes	121 (24.5%)
No	373 (75.5%)
Missing	0 (0.0%)
Complications (Clavien-Dindo)	
Grade I	78 (15.8)
Grade II	156 (31.6%)
Grade III	30 (6.1%)
Grade IV	7 (1.4%)
Missing	0 (0.0%)
Postoperative Adjuvant Therapy	
Yes	159 (32.2%)
No	335 (67.8%)
Missing	0 (0.0%)
Tumor regression grade	
TRG 0	153 (31.0%)
TRG 1	89 (18.0%)
TRG 2	186 (37.7%)
TRG 3	66 (13.4%)
Missing	0 (0.0%)

LVI, lymphovascular invasion; PNI, peripheral nerve invasion; TRG, tumor regression grade.

### Characteristics associated with TRG

Patients were divided in to two groups (TRG 0–1 and TRG 2–3) for comparison. The analysis of characteristics associated with TRG was showed in Table 2. The TRG score is significantly associated with smoke history (*p* = 0.02), LVI and/or PNI (*p* < 0.01), and postoperative adjuvant therapy (*p* < 0.01). Meanwhile, tumor characteristics including tumor length (*p* < 0.01) and tumor differentiation grade (*p* < 0.01) are also significantly associated with TRG scores. Patients with poor response to neoadjuvant chemoradiotherapy (TRG2–3) were more likely to have: smoke history, longer tumor length, poorer tumor differentiation grade, poorer tumor stage, more positive lymph nodes, advanced stage, lymphovascular and peripheral nerve invasion.

**Table 2:** Patient characteristics associated with tumor regression grade.

Variables	TRG 0–1 (*n* = 242) No. (%)	TRG 2–3 (*n* = 252) No. (%)	*p*-Value
Gender			0.13
Male	191 (78.9%)	213 (84.5%)	
Female	51 (21.1%)	39 (15.5%)	
Missing	0 (0.0%)	0 (0.0%)	
Age (year)			0.12
≤ 70	202 (83.5%)	223 (88.5%)	
> 70	40 (16.5%)	29 (11.5%)	
Missing	0 (0.0%)	0 (0.0%)	
Smoke			0.02
Yes	111 (45.9%)	141 (56.0%)	
No	131 (54.1%)	109 (43.3%)	
Missing	0 (0.0%)	2 (0.8%)	
Tumor site			0.18
Upper	24 (9.9%)	37 (14.7%)	
Middle	110 (45.5%)	118 (46.8%)	
Lower	108 (44.6%)	97 (38.5%)	
Missing	0 (0.0%)	0 (0.0%)	
Tumor length (cm)			0.00
≤ 3 cm	187 (77.3%)	121 (48.0%)	
> 3 cm	55 (22.7%)	131 (52.0%)	
Missing	0 (0.0%)	0 (0.0%)	
ypTNM			0.00
I	194 (80.2%)	39 (15.5%)	
II	6 (2.5%)	69 (27.4%)	
IIIA	30 (12.4%)	25 (9.9%)	
IIIB	8 (3.3%)	108 (42.9%)	
IVA	4 (1.7%)	9 (3.6%)	
Missing	0 (0.0%)	0 (0.0%)	
ypT			0.00
T0	161 (66.5%)	6 (2.4%)	
T1	47 (19.4%)	23 (9.1%)	
T2	24 (9.9%)	48 (19.0%)	
T3	10 (4.1%)	175 (69.4%)	
Missing	0 (0.0%)	0 (0.0%)	
ypN			0.00
N0	199 (82.2%)	109 (43.3%)	
N1	34 (14.0%)	88 (34.9%)	
N2	5 (2.1%)	44 (17.5%)	
N3	4 (1.7%)	9 (3.6%)	
Missing	0 (0.0%)	0 (0.0%)	
Tumor differentiation			0.00
G1-2	32 (13.2%)	101 (40.1%)	
G3	28 (11.6%)	133 (52.8%)	
Gx	182 (75.2%)	18 (7.1%)	
Missing	0 (0.0%)	0 (0.0%)	
LVI and/or PNI			0.00
Yes	10 (4.1%)	111 (44.0%)	
No	232 (95.9%)	141 (56.0%)	
Missing	0 (0.0%)	0 (0.0%)	
Postoperative adjuvant therapy			0.00
Yes	55 (22.7%)	104 (41.3%)	
No	187 (77.3%)	148 (58.7%)	
Missing	0 (0.0%)	0 (0.0%)	

TRG, tumor regression grade; LVI, lymphovascular invasion; PNI, peripheral nerve invasion.

### Survival analysis

**Figure 2:**
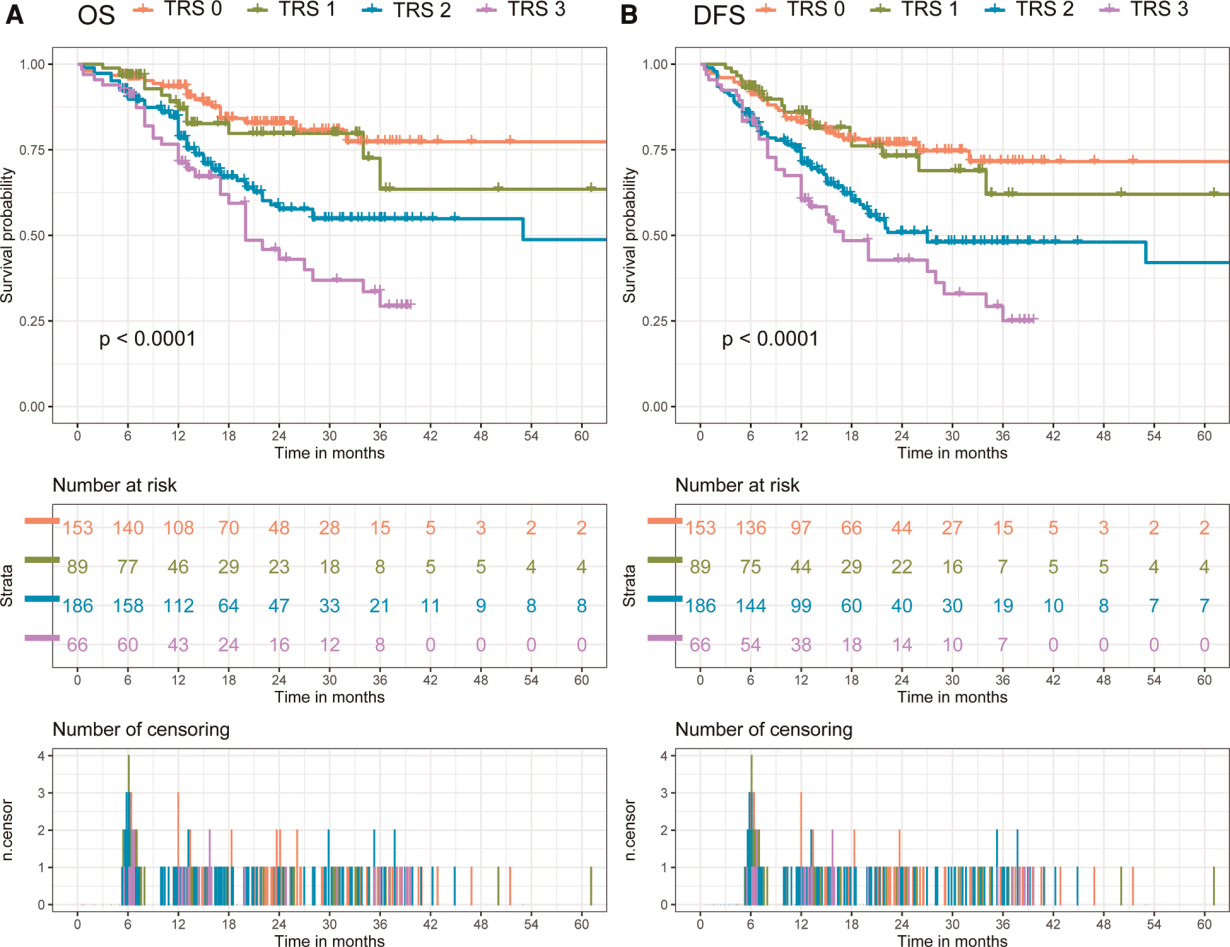
Comparison of overall survival (OS) and disease-free survival (DFS) in all cohort. (**A**) Comparison of OS between patients with different tumor regression grade. (**B**) Comparison of DFS between patients with different tumor regression grade.

**Figure 3:**
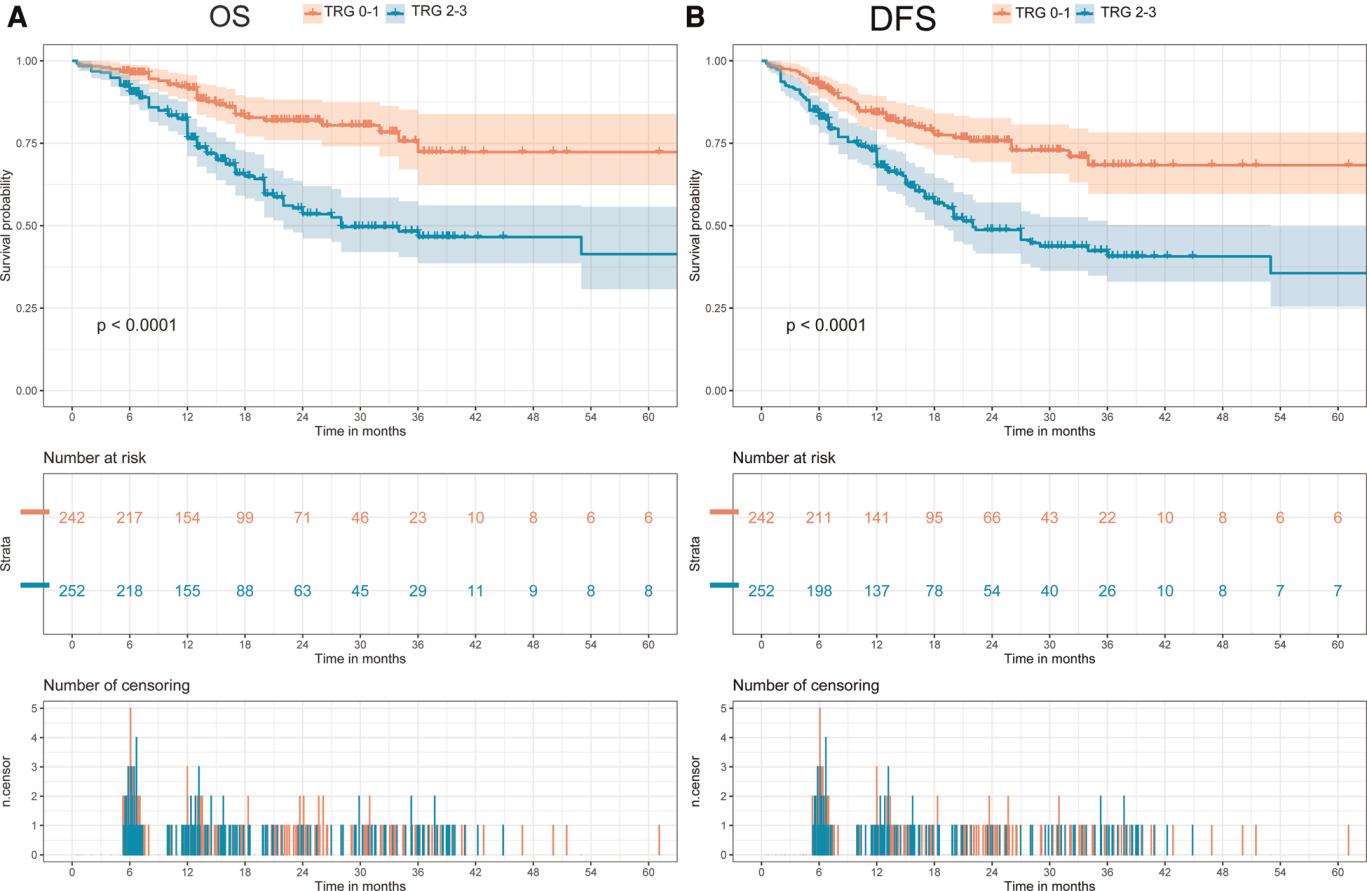
Comparison of overall survival (OS) and disease-free survival (DFS) between two groups (TRG 0-1 vs. TRG 2-3). (**A**) Comparison of OS between two groups (TRG 0-1 vs. TRG 2-3). (**B**) Comparison of DFS between two groups (TRG 0-1 vs. TRG 2-3).

The median follow-up was 13.6 months (interquartile range 6.9–24.7 months) for the overall cohort. In all cohort, the OS rate was 81.8% (95% CI: 78.1–85.5%) after 1 year, 58.7% (51.8–65.6%) after 3 years, and 54.8% (45.0–64.6%) after 5 years. Meanwhile, the DFS rate was 75.8% (71.7–80.0%) after 1 year, 53.4% (46.9–59.9%) after 3 years, and 54.8% (39.5–70.1%) after 5 years. When comparing patients with different TRG, patients with poorer response had a significantly shorter post-resection OS and DFS compared with those with better response (Log-Rank, OS: *p* < 0.01; DFS: *p* < 0.01, Figure 2). Patients were then divided in to two groups (TRG 0–1 and TRG 2–3) for comparison. The OS and DFS of patients with poor response (TRG 2–3) were significantly shorter than those with complete response (TRG 0–1) (Figure 3).

### Cox regression analysis

The results of univariate and multivariate Cox regression were showed in Tables 3, 4. The ypTNM stage and 3 tumor characteristics including tumor length, tumor differentiation grade and TRG were included for univariate Cox regression. The results showed that the ypTNM stage and 3 tumor characteristics were all significantly associated with OS and DFS. Patients with worse OS and DFS were more likely to have: longer tumor length, poorer tumor differentiation grade, poorer TRGs, and more advanced ypTNM stage. These four variables were selected for multivariate Cox regression model entry due to *p* < 0.05 on univariate analysis. The results of Cox regression analysis on OS shows that only ypTNM stage are independently prognostic factor for OS in patients undergoing trimodal therapy. The results of Cox regression analysis on DFS shows that both ypTNM stage and tumor length were independently prognostic factors for DFS. However, TRG is not an independently prognostic factor for OS (*p* = 0.922) or DFS (*p* = 0.526).

**Table 3:** Impact of treatment outcome and prognostic relevance on overall survival and disease-free survival.

Univariate analyses	3-year OS % (95% CI)	Overall survival		3-year DFS % (95% CI)	Disease-free survival	
Variables		HR (95% CI)	*p*-Value		HR (95% CI)	*p*-Value
ypTNM stage			0.000			0.000
I	79.9 (70.5–89.3)	1 (ref)		74.4 (66.2–82.6)	1 (ref)	
II	67.0 (52.5–81.5)	2.193 (1.184–4.064)		62.7 (48.4–77.0)	1.730 (1.017–2.941)	
IIIA	56.5 (37.9–75.1)	3.343 (1.802–6.202)		52.2 (32.4–72.0)	2.143 (1.224–3.750)	
IIIB	28.6 (16.8–40.4)	5.285 (3.300–8.466)		21.7 (11.1–32.3)	4.266 (2.874–6.333)	
IVA	0.0 (0.0–0.0)	15.708 (7.592–32.499)		0.0 (0.0–0.0)	13.676 (7.034–26.592)	
Tumor length (cm)			0.000			0.000
≤ 3	68.2 (58.6–77.8)	1 (ref)		63.5 (54.9–72.1)	1 (ref)	
> 3	47.1 (37.7–56.5)	2.246 (1.572–3.210)		41.0 (31.8–50.2)	2.152 (1.567–2.955)	
Tumor differentiation			0.000			0.000
G1-2	56.6 (43.5–69.7)	1 (ref)		50.2 (63.1–63.1)	1 (ref)	
G3	43.9 (32.7–55.1)	1.703 (1.119–2.592)		37.5 (26.7–48.3)	1.595 (1.095–2.324)	
Gx	73.4 (64.8–82.0)	0.605 (0.375–0.979)		72.7 (64.1–81.3)	0.590 (0.386–0.902)	
Tumor regression grade			0.000			0.000
TRG 0	95.2 (91.7–98.7)	1 (ref)		71.4 (60.8–82.0)	1 (ref)	
TRG 1	63.5 (41.2–85.8)	1.259 (0.640–2.476)		62.0 (43.8–80.2)	1.058 (0.591–1.894)	
TRG 2	54.9 (45.3–64.5)	2.432 (1.488–3.975)		48.0 (38.6–57.4)	2.045 (1.345–3.107)	
TRG 3	29.3 (14.0–44.6)	3.790 (2.201–6.527)		25.1 (10.4–39.8)	3.042 (1.888–4.902)	

OS, overall survival; DFS, disease-free survival; TRG, tumor regression grade.

**Table 4:** The multivariate analysis of overall survival and disease-free survival.

Multivariate analyses	Overall survival			Disease-free survival		
Variables	HR	95% CI of HR	*p*-Value	HR	95% CI of HR	*p*-Value
ypTNM stage			0.000			0.000
II vs. I	2.074	0.957–2.120		1.586	0.786–3.200	
IIIA vs. I	3.139	1.588–6.204		2.014	1.086–3.735	
IIIB vs. I	5.222	2.709–10.066		4.097	2.234–7.516	
IVA vs. I	11.804	4.803–29.010		15.708	7.592–32.499	
Tumor length (cm)			0.067			0.025
> 3 vs. ≤ 3	1.439	0.976–2.120		1.485	1.051–2.099	
Tumor differentiation			0.149			0.114
G3 vs. G1-2	1.535	0.996–2.365		1.416	0.963–2.082	
Gx vs. G1-2	1.264	0.672–2.378		0.913	0.494–1.687	
Tumor regression grade			0.922			0.526
TRG1 vs. TRG0	0.763	0.355–1.643		0.610	0.308–1.207	
TRG2 vs. TRG0	0.845	0.354–2.017		0.670	0.312–1.439	
TRG3 vs. TRG0	0.829	0.324–2.124		0.605	0.262–1.396	

TRG, tumor regression grade.

### Building and validating the novel nomogram

Multivariate Cox proportional hazards model by using a backwards model selection procedure was utilized to analyze the factors with a *P*-value < 0.05 in univariate analysis. Finally, factors including ypTNM stage and tumor length were identified as independent predictors of DFS and were included in the predictive model (Supplementary Table S1). The predictive model was virtually presented in the form of a nomogram (Supplementary Figure S1). The C-index of the novel nomogram was 0.702, reflecting the good discrimination ability of the model.

## Discussion

Esophagectomy with radical lymphadenectomy is the primary treatment for localized ESCC. Recently, preoperative chemoradiation has become the standard treatment among most patients with potentially curable ESCC, since the CROSS Group reported good results of neoadjuvant therapy (14, 15). Therefore, concurrent neoadjuvant chemoradiotherapy followed by surgery has been considered as a preferred treatment strategy for these patients diagnosed as ESCC in China. Many systematic reviews concluded that preoperative chemoradiation could be an effective treatment for locally advanced esophageal cancer, since it reduces margin-positive resections and improves survival rates (16). Recently, tumor regression grade has been introduced to evaluate the efficacy of neoadjuvant therapy (9). Complete pathologic response to neoadjuvant therapy has been proved to be associated with higher survival rates and lower recurrence rates and is, therefore, a vital prognostic factor.

Many scoring systems have been proposed to evaluate pathologic response. Mandard et al. (17) first reported a five-tier system for assessing TRG in esophageal carcinoma in 1994. Subsequent studies validated its efficacy of predicting long-term survival. Afterwards, Chirieac et al. (18) introduced a three-tier system in 2005 and Schneider et al. (19) published a four-tier system that considers lymph node involvement. Each one of these systems emphasizes determinate histological features, evaluating the presence/absence of residual cancer cells differently. In the same year, Ryan et al. (10) reported a practical three-point system to assess TRG of patients with locally advanced rectal adenocarcinoma who underwent neoadjuvant therapy. Compared with other systems, it is associated with better reproducibility and more concordance between pathologists. The use of Ryan scoring system for ESCC and its correlation with OS, DFS, and recurrence of disease is currently unprecedented (11). Ryan scoring system enables easier and more clear-cut scoring than other scoring systems and can predict long-term survival and recurrence.

Takeda et al. (11) in 2019 first introduced Ryan scoring system to evaluate the efficacy of neoadjuvant therapy and explore its correlation with survival outcomes. They used a three-tier system, in which score 1 was defined as complete response (no viable cancer cells) or near-complete response (single cells or rare small groups of cancer cells). Their study concluded that Ryan score predicts survival and recurrence rates. However, several limitations existed in their study. Three-tier system could not precisely stratify the EC patients undergoing trimodal therapy. Therefore, in our study the modified Ryan scoring system (a four-tier system) was evaluated for prognosis. In this system, the Score 1 was divided into two scores: TRG 0 (complete response) and TRG 1 (near complete response). On the other hand, the study by Takeda et al. (11) only used univariable Cox regression modal to evaluate the prognostic value of Ryan scoring system. Therefore, whether Ryan scoring system could be an independently prognostic factor for EC patients remains unclear. The results of our study showed that modified Ryan scoring system is not an independently prognostic factor for OS or DFS in ESCC patients undergoing trimodal therapy. Furthermore, only ESCC patients were included in our study, which is different from the study by Takeda et al. in which ESCC and EAC patients were both included.

The primary purpose of this study was to evaluate the prognostic impact of TRG after preoperative chemoradiotherapy on OS and DFS in ESCC patients. The secondary aim of this study was to assess the prognostic impact of tumor characteristics including tumor length and tumor differentiation on OS and DFS. To our knowledge, this is the first study based on 8^th^ AJCC ypTNM staging and modified Ryan scoring system to investigate the prognostic impact of tumor characteristics including tumor length, tumor differentiation, and TRG on OS and DFS in ESCC patients undergoing trimodal therapy.

The results of the present study showed that smoke status and tumor length of patients could influence the pathologic response to neoadjuvant chemoradiotherapy. Patients who had smoke history were more likely to have poor response to neoadjuvant therapy. When the tumor length of patients was more than 3 cm, the risk of poor response also increased. Hollis et al. (8) conducted a retrospective analysis including 358 patients and found that tumor size is associated with tumor grade, pathological T and N stages, and prognosis. Several previous studies on gastric cancer have also shown that tumor size is related to TRG and prognosis (20–22), but the mechanism has not been investigated. Meanwhile, the results showed that TRG was not only correlated with the tumor invasion status after neoadjuvant CRT, but also associated with lymph node metastasis. The proportion of ypN+patients in TRG 2–3 group were significantly higher than that in TRG 0–1 group. This result indicated that neoadjuvant chemoradiotherapy could concurrently improve the status of lymph node metastasis in patients with complete or near complete response. Remarkably, in patients undergoing neoadjuvant chemoradiotherapy, TRG was significantly correlated with incidence of LVI and/or PNI. Numerous reports have demonstrated that LVI and PNI are poor prognostic factors for patients with ESCC who have undergone surgery. The present study indicated that patients with complete response were less likely to have LVI and PNI, which implied that neoadjuvant chemoradiotherapy could also be an effective treatment to reduce the LVI and PNI of ESCC patients. In general, the purpose of neoadjuvant chemoradiotherapy is not only to shrink the primary tumor, but also to prevent the early spread of systemic disease.

The results of our study showed that TRG at the primary site were significantly correlated with systemic therapeutic effects, including a better survival outcome and a reduction in recurrence. Better long-term survival was observed in patients with complete or near complete response. Meanwhile, the univariable Cox regression analysis indicated that TRG could be a prognostic factor for OS and DFS. However, this prognostic effect was eliminated by the ypTNM stage in multivariable Cox regression analysis, which indicated that TRG was strongly associated with ypTNM stage. Therefore, TRG is not an independently prognostic factor for OS and DFS in ESCC patients undergoing trimodal therapy.

Tumor length was the only independently prognostic factor for DFS in tumor characteristics. Patients with tumor length > 3 cm had a 40% increased risk of death and recurrence compared with patients with tumor length ≤ 3 cm (HR: 1.413, 95% CI: 1.006–1.985, *p* = 0.046). The results implied that the extent of tumor invasion is also an important prognostic factor in addition to ypT stage, which may also be included in the ypTNM staging system. The C-index of the novel nomogram was 0.702, reflecting the good discrimination ability of the model.

In addition to tumor characteristics, perioperative complications are also an important factor affecting the postoperative prognosis of patients with esophageal cancer (23, 24). Multidisciplinary management of perioperative complications remains an important way to improve the long-term prognosis of patients.

There are several limitations inherent to the retrospective and observational nature of this study design to be considered. Meanwhile, this study is a single-center research, which may lead to selection bias. Therefore, controlled prospective studies, with multi-center samples are warranted to validate modified Ryan scoring system and evaluate its concordance for ESCC. Furthermore, future studies should evaluate different radiation field setting and different neoadjuvant regimens other than taxane and platinum based.

## Conclusions

This study evaluated the prognostic value of modified Ryan scoring system for ESCC after trimodal therapy and concluded that modified Ryan scoring system can predict survival and recurrence rates but is not an independently prognostic factor for OS and DFS. The smoke status, tumor length, status of LVI and PNI, and ypN stage are significantly correlated with TRG score. Tumor length is an independently prognostic factor for DFS in ESCC patients undergoing neoadjuvant chemoradiation.

## Data Availability

The original contributions presented in the study are included in the article/**Supplementary Material**, further inquiries can be directed to the corresponding author/s.

## Supplementary material

Supplementary Figure S1 Nomogram predicting the disease-free survival (DFS) for patients undergoing neoadjuvant chemoradiotherapy and esophagectomy. For every patient, 2 lines are drawn upward to determine the points received from the 2 predictors in the nomogram. The sum of these points is located on the ‘Total Points’ axis. In addition, a line is drawn downward to determine the possibility of 12-, 24-, 48-, and 60-month DFS, and the median DFS for patients with the same total score.

Supplementary Table S1 Multivariate Cox regression model using a backwards model selection procedure.
