# Supplementary material for: Prognostic significance of tumor regression grade in esophageal squamous cell carcinoma after neoadjuvant chemoradiation
Source: Front Surg. 2023 Jan 6;9:1029575. doi: 10.3389/fsurg.2022.1029575 (PMC9852042; doi:10.3389/fsurg.2022.1029575)
Supplement: Supplementary Table S1 — Multivariate Cox regression model using a backwards model selection procedure. [file Table1.doc]

Supplementary Table 1: Multivariate Cox regression model using a backwards model selection procedure

| Multivariate analyses | Progression-free survival | | |
| --- | --- | --- | --- |
| Variables | HR | 95% CI of HR | P-value |
| ypTNM stage |  |  | 0.000 |
| II versus I | 1.565 | 0.912-2.687 |  |
| IIIA versus I | 2.075 | 1.185-3.635 |  |
| IIIB versus I | 3.795 | 2.514-5.728 |  |
| IVA versus I | 10.827 | 5.374-21.811 |  |
| Tumor length (cm) |  |  | 0.046 |
| > 3 versus ≤ 3 | 1.413 | 1.006-1.985 |  |
